# Moebius Syndrome: What We Know So Far

**DOI:** 10.7759/cureus.35187

**Published:** 2023-02-19

**Authors:** Syed Muhammad Hussain Zaidi, Izna Najam Syed, Umair Tahir, Tayyaba Noor, Muhammad Saad Choudhry

**Affiliations:** 1 Internal Medicine, Dow University of Health Sciences, Civil Hospital Karachi, Karachi, PAK; 2 Internal Medicine, Rawalpindi Medical University, Karachi, PAK; 3 General Surgery, Dow University of Health Sciences, Civil Hospital Karachi, Karachi, PAK

**Keywords:** congenital ophthalmoplegia and facial paresis, moebius congenital oculofacial paralysis, moebius spectrum, moebius sequence, moebius syndrome

## Abstract

Moebius syndrome (MBS) is a rare congenital cranial nerve disorder characterized by unilateral, bilateral symmetrical, or asymmetrical facial (VII) and abducens (VI) nerve palsies. Genetics and rhombencephalon vascular disturbances from intrauterine environmental exposures have been attributed to its development. It can present with various orofacial abnormalities. Although the diagnosis is purely clinical, certain characteristic features are present in the brain's images. With no cure, it is essential to devise management on a personalized basis. We discuss etiology, presentation, diagnostic approaches, and effective management in the existing literature. This comprehensive review examines the clinic-pathological aspects of Moebius syndrome. The authors employed the PUBMED base index to identify pertinent literature and reference it according to research keywords. Findings suggest the most popular etiology is the theory of intrauterine vascular disruption to the brainstem during embryogenesis, followed by the genetic hypothesis. Intrauterine environmental exposures have been implicated as potential risk factors. Facial and abducens nerve palsies are the most common presenting features. However, clinical manifestations of lower cranial nerves (IX, X, XI, XII) may be present with orthopedic anomalies and intellectual deficiencies. The diagnosis is clinical with minimal defined diagnostic criteria. Characteristic radiological manifestations involving the brainstem and cerebellum can be observed in imaging studies. With no definitive treatment options, a multidisciplinary approach is employed to provide supportive care. Despite radiological manifestations, Moebius syndrome is diagnosed clinically. Although incurable, a multidisciplinary approach, with personalized rehabilitative measures, can manage physical and psychological deficits; however, standard guidelines need to be established.

## Introduction and background

Moebius syndrome (MBS) is a rare, congenital condition characterized by a disorder of the cranial nerves involved in the control of the muscles of the face and eyes, leading to several orofacial and ocular defects, along with associated limb abnormalities [1,2]. It also shows a certain degree of overlap with various other congenital syndromes with facial involvement and cranial nerve involvement [3]. The diagnosis of Moebius syndrome is based largely on the clinical features that are mostly the result of muscular abnormalities arising from nerve defects [3], and despite the lack of a specific diagnostic test, certain findings on brain imaging can be confidently attributed to the syndrome [4-6]. The disease is mostly sporadic and has a highly variable incidence, yet studies have recognized some genetic patterns that may predispose to the origin of the syndrome [2,7]. Although the etiology of the disorder is unclear, two hypotheses have been postulated recently, suggesting that neuromuscular defects have either a genetic or a vascular origin [2,8]. It often has congenital facial weakness and is combined with abnormal ocular abduction [9]. A definitive cure for MBS is unavailable, and therefore treatment strategies rely upon the management of the resulting disorders either conservatively or surgically [10]. Fetal toxin exposure to several substances has also been noted to contribute toward the abnormality [11,12]. This review article aims to discuss the etiological basis and clinical manifestations.

## Review

Methodology

The articles were researched from the PUBMED database by using the search keywords including "Moebius syndrome", "facial nerve" "congenital weakness", and "orofacial abnormalities". Eligibility was determined on basis of articles comprising the pathophysiology, etiology, treatment, and management of Moebius syndrome. No automation process was used. Papers were reviewed and selected manually. Papers were excluded if the baseline orofacial abnormalities were from different congenital disorders or were cross-sectional surveys. Research articles were reviewed by independent authors for eligibility and exclusion confirmation.

Etiology

Although the exact etiology and pathogenesis of Moebius syndrome are not known, several theories have been postulated including genetic causes and intrauterine environmental exposures. Currently, a hypoxic-ischemic insult to the brainstem during embryogenesis is the most prevalent etiology of Moebius syndrome. Disruption of vascular supply to the territory of the subclavian artery during the sixth week of embryological development may result in anomalous development of the brainstem [13]. This vascular disturbance of the subclavian artery may be due to either fetal vascular events like thrombus formation, embolism, and hemorrhage, or vasoconstriction as a result of maternal use of abortifacients like misoprostol and maternal cocaine abuse [11,14]. Owing to the distribution pattern of the embryonic basilar artery (branch of the vertebral artery), the dorsomedial aspect of the brainstem is the region most susceptible to a hypoxic-ischemic insult [15].

The postmortem histopathological assessment of brainstems with the Moebius sequence further corroborates the likelihood of an underlying vascular pathology due to the presence of features of focal necrosis with regional gliosis and calcification within the arterial territories of the brainstem [13]. However, the involvement of the subclavian artery does not account for the clinical manifestations involving the abducens and facial nerves in patients with Moebius syndrome, as these structures do not derive their blood supply from the subclavian artery [10].

Role of genetics

Another widely acknowledged hypothesis is the genetic etiology of Moebius syndrome. Two genetic loci have been reported in association with Moebius syndrome, at 3q21-q22 and 10q. It is mentioned in the Online Mendelian Inheritance in Man (OMIM) number 15700, with a gene map locus of 13q12.2-q13. Additionally, de novo mutations involving the REV3L and PLXND1 genes have also been observed in association with congenital facial palsy in Moebius syndrome [12,16]. Moreover, mutations of the HOXA1, HOXB1, and TUBB3 genes have been observed in atypical forms of Moebius syndrome [17-19]. In rare instances, familial patterns have been identified as well, being autosomal dominant. However, they do not account for the majority of the cases [20].

Other potential etiological determinants of Moebius syndrome include intrauterine infections, hyperthermia, intrauterine trauma, and prenatal exposure to teratogens like alcohol, thalidomide, ergotamine, and benzodiazepines [21].

Epidemiology

The occurrence of Moebius syndrome is rare, and there are very limited data available regarding its epidemiology. Studies estimate the prevalence to be 0.0002% - 0.002% of live births [7,22]. Moreover, no difference has been found regarding the incidence among different genders or races [22,23].

Clinical features

The wide array of clinical features seen in the syndrome arises due to the involvement of several cranial nerves, particularly CN VI and CN VII, which control the functions of the eye and face, respectively [1]. This mainly gives rise to various orofacial abnormalities, as well as ocular deformities [2]. Other features include developmental delay, musculoskeletal malformations, neurological disorders, mental retardation, and problems with the endocrine and respiratory systems [1,24]. The major abnormalities are included in the CLUFT grading system, which relies on the cranial nerve, facial, upper and lower limbs, and thoracic findings to grade the disease (Table 1) [25].

**Table 1:** CLUFT grading system CLUFT: cranial nerve, facial, upper and lower limbs, and thoracic findings

	FEATURE	GRADE
C:	Cranial Nerves	
	CN-VII (Partial)	0
	CN-VI and CN-VII (Partial)	1
	CN-VI and CN-VII (Complete)	2
	Additional Nerve Involvement	3
	If Bilateral and Equal Add	B
L:	Lower Extremity	
	Normal	0
	Talipes Equinovarus, Syndactyly, Ankylosis	1
	Absent Phalanges	2
	Longitudinal or Transverse Deficits	3
U:	Upper Extremity	
	Normal	0
	Digital Hypoplasia or Failure of Differentiation	1
	Ectrodactyly	2
	Failure of Formation, Longitudinal or Transverse	3
F:	Facial Structural Anomaly	
	Normal	0
	Cleft Palate	1
	Micrognathia	2
	Microtia, Microphthalmia, Abnormal Joint, etc.	3
T:	Thorax	
	Normal	0
	Scoliosis	1
	Pectoral Hypoplasia/Breast Anomaly	2
	Chest Wall Deformity, Breast or Pectoral Aplasia	3

The most common of presentations are those due to the abnormalities of the facial and abducens nerves. Among the orofacial deformities, the most common is facial palsy due to CN VII involvement, which is usually bilateral but incomplete, giving rise to altered abilities of facial sensation, speech, feeding, and expression [3]. Moreover, cleft lip and palate, micrognathia, maxillary underdevelopment, hypertelorism, aglossia/microglossia, and dental problems have also been noted [26,27]. Also quite common is the involvement of CN VI, leading to ocular manifestations, such as strabismus, ophthalmoplegia, lateral gaze palsies, and ptosis, which can further result in exposure keratopathy [28]. Issues originating due to other cranial nerve palsies can further create problems related to swallowing and breathing as a result of palatopharyngeal dysfunction, inadequate cough reflex, glottic spasm, and laryngeal paralysis [2,29]. The musculoskeletal anomalies range from hypoplasia to aplasia of the digits of upper and lower limbs, brachydactyly, syndactyly, ectrodactyly, and several other orthopedic conditions such as talipes equinovarus, ankylosis, and scoliosis [2,25,28,30]. Occasionally, there is an absence or hypoplasia of the pectoralis major as part of the Poland syndrome [3].

Although the intelligence of these patients is usually comparable to that of the normal population, a few studies do show an association, to some degree, with behavioral alteration, autism, and intellectual disability [23,31]. Another notable association is with trigeminal trophic syndrome (trigeminal anesthesia, facial paresthesia, facial ulceration), which can arise due to congenital syndrome [32]. In addition to the already mentioned Poland syndrome, Moebius syndrome also has a significant association with a number of other clinical syndromes like cerebral palsy, Kallmann syndrome, Pierre Robin sequence (micrognathia, falling-back tongue, respiratory difficulty), Klippel-Feil anomaly (fusion of cervical vertebrae), Melkersson-Rosenthal syndrome (granulomatous cheilitis, facial palsy, tongue fissuring), and various congenital myopathies [3,9,25,33].

Investigations and diagnostic criteria

The diagnosis of Moebius syndrome is mostly on the basis of clinical criteria; although genetic testing exists, it is expensive and not accessible, following the exclusion of syndromes with similar clinical manifestations [10]. However, in the absence of definitive diagnostic criteria, the clinical assessment and diagnosis of patients with the condition was a challenge for medical practitioners. To remedy this diagnostic dilemma, a definitive diagnostic criterion for Moebius syndrome was formulated in 2007 by a group of clinical researchers at the Moebius Syndrome Foundation research conference in Maryland, USA. Currently, the minimal diagnostic criteria of Moebius syndrome include 1) Congenital, non-progressive symmetrical or asymmetrical, unilateral, or bilateral facial palsy that is lower motor neuron type in nature, 2) Unilateral or bilateral symmetrical or asymmetrical abducens nerve palsy. Both of these criteria should be met for the diagnosis of Moebius syndrome [34].

Although the diagnosis of Moebius syndrome almost exclusively depends mostly on the patient’s symptomatology and clinical features, findings on radiological imaging of the brain can play a pivotal role in excluding similar pathologies. The radiological findings on the CT brain of the patients with Moebius syndrome include calcification of the brainstem at the site of ischemic necrosis, most frequently the dorsal aspect of the pons, in the region of the abducens nerve. Other CT manifestations of clinical significance include medial deviation of the eyes, hypoplasia or dysplasia of the brainstem, and cerebellar hypoplasia. Similarly, the MRI brain of patients with Moebius syndrome shows calcification of the pons in the area of the abducens nerve, hypoplasia of the cerebellum, and a hypoplastic or atrophic brainstem along with straightening of the floor of the fourth ventricle, which indicates the absence of facial colliculus [5,6,35].

As stated earlier, the diagnosis of Moebius syndrome is made after excluding similar syndromes. One such syndrome is the Melkerrson-Rosenthal syndrome, which manifests as a classic triad of congenital facial palsy, tongue fissuring, and lip swelling. Another clinically significant differential diagnosis of Moebius syndrome is Poland syndrome. Patients with Poland syndrome present with congenital palsies of the facial and abducens nerves along with ipsilateral hypoplasia of the pectoralis muscle. Other conditions that need to be ruled out before making the diagnosis of Moebius syndrome include hereditary congenital facial palsy (HCFP) (characterized by isolated facial palsy without the involvement of the abducens nerve), Duane retraction syndrome (characterized by a limited abduction or adduction of the eye along with an inward retraction of the eyeball and shortening of palpebral fissure on horizontal eye movements), congenital fibrosis of the extraocular muscles (presents with severe congenital strabismus, ptosis, and vertical gaze palsy), DiGeorge syndrome, and CHARGE syndrome [14,28,36-38].

Management

The management of Moebius syndrome is through a multidisciplinary supportive approach that is aimed at addressing the various symptoms associated with the condition since no definitive treatment exists for MBS. A multitude of healthcare professionals should be involved in the management of patients with Moebius syndrome, including primary care physicians, neurologists, orthopedic surgeons, otolaryngologists, ophthalmologists, plastic and reconstructive surgeons, medical geneticists, dentists, psychologists, speech therapists, physical therapists, and occupational therapists [39]. One of the most common manifestations of MBS is sucking and feeding difficulties among infants. Reduced lip seal as a result of a cleft lip leads to difficulties retaining food in the oral cavity, a small tongue causes problems with the propulsion of the food bolus posteriorly toward the oropharynx and lower cranial nerve palsies (IX, X, XI) and may lead to neuromuscular incoordination while swallowing. Early input from clinical dieticians, regular meticulous monitoring for postnatal weight gain, and evaluation for swallowing are crucial in the management of MBS. Moreover, these infants may benefit from the use of specialized bottles such as Haberman’s feeder. In severe cases, additional nutritional support may be required via feeding tubes [10,39].

Global developmental delay is not a common feature of MBS. On the contrary, patients with the condition mostly have speech and language delays while some may have poor motor coordination [10]. Consequently, early initiation of speech and physical rehabilitation therapies is recommended to improve speech, language, swallowing, and motor skills and coordination [39]. Problems with sucking and feeding among infants, in particular, should prompt an early audiology and speech therapy assessment and rehabilitation. The early pre-speech intervention leads to optimal conditions for speech and language development later in life [10,22,40]. Some children with MBS may develop problems associated with mastication at a later point in life. This is due to a combination of weak bite, structural abnormalities involving the tongue, and oromandibular hypoplasia. However, in most cases, patients develop compensatory mechanisms to counteract these problems [22]. Furthermore, specialized exercises designed to target oral musculature may improve the sense of awareness of particular groups of facial muscles. They may be particularly beneficial for older children and adults and may help strengthen facial movement. “The smile operation” or facial reanimation surgery is a relatively new surgical procedure aimed at improving facial movements. This surgical intervention enables the patient to smile and allows the achievement of facial symmetry. Moreover, it facilitates speech by reducing problems associated with pronunciation and swallowing difficulties along with improving the dental health of the patient. Variable techniques are employed in the smile operation, but the most frequently used technique involves the microvascular transfer of the gracilis muscle to the face with the use of the nerves innervating the masseter. Transfer of the temporalis tendon and bilateral selective neurolysis are other techniques used in facial reanimation surgery [38,39].

One of the manifestations of Moebius syndrome is abnormal ocular mobility. This includes strabismus, Duane’s retraction syndrome, and conjugate gaze palsies. Moreover, incomplete closure of the eyelids due to a relative imbalance between the functions of orbicularis oculi (eye closure) and levator palpebrae superioris (eye-opening) may lead to corneal ulcerations and, ultimately, blindness. Therefore, an early ophthalmological assessment is recommended with the goal being the prevention of corneal ulceration as well as the diagnosis and rectification of gaze palsies. Injection of botulinum toxin into the medial rectus muscle can prevent the development of contractures, thereby facilitating the achievement of conjugate gaze. Moreover, it can be beneficial in subsequent surgery for the correction of strabismus [22]. Other therapeutic modalities, such as eye surgeries (tarsorrhaphy or gold weights), lubricant ointments, eye drops, or taping, to prevent exposure keratopathy can be utilized for the management of lagophthalmos associated with Moebius syndrome [10,39,41]. Another major concern associated with MBS is the patient’s mental health due to the social stigma surrounding the condition due to a lack of public awareness about it. Moreover, individuals with Moebius syndrome may struggle with developing and maintaining social relationships and a positive body image. These challenges can be overcome by healthcare professionals actively listening to the patients during their interactions, deriving social support from local charities, and employing cognitive behavioral therapy as well as social skills training to hone the patient’s abilities to communicate in a social setting [42].

Treatment

No definitive treatment exists for Moebius syndrome, as is the case for any congenital neurological disease. However, individuals with Moebius syndrome who do not develop any significant life-threatening complications during the first year of their life enjoy normal life expectancy with appropriate medical care aimed at specific neurological and structural deficits [43]. Moreover, the enhancement of motor and sensory abilities via early rehabilitation along with support from parents can further improve the long-term physical and psychological prognosis of children with Moebius syndrome [2]. Social stigmatism against neurological disorders with visible signs, leading to a general lack of support from people, especially parents, can be devastating for the psychological state of children with Moebius syndrome [42]. Ora-facial and limb deformities can be corrected through surgical interventions and may improve rehabilitation. Due to orofacial abnormalities, Infants often require feeding assistance and nutritional management. Physical, occupational, and speech therapy is essential in rehabilitation. Ophthalmic abnormalities such as Strabismus and lagophthalmos are surgically correctable. Temporalis tendon transfer and bilateral selective neurolysis are newer surgical techniques involved in management [38]. Although the patient might have related infections from underlying malnutrition and orofacial abnormalities, it is prudent to screen for underlying immunodeficiencies and be evaluated by an immunologist with a low threshold for recurrent infections, as a case report has identified X-linked Agammaglobulinemia and Moebius syndrome [44].

## Conclusions

In conclusion, the diagnosis of Moebius syndrome is exclusively clinical with laboratory investigations being unwarranted in the diagnosis of the condition, despite the characteristic radiological manifestations. Like any other congenital neurological condition, Moebius syndrome is incurable. However, a multidisciplinary approach can be used to manage the various physical and psychological deficits associated with the disease, but standard guidelines need to be established in this regard. Furthermore, the rehabilitative approaches should be personalized to each patient and should be updated regularly following the patient’s functional assessment.
